# The impact of VEGF signalling pathway inhibitors and/or immune checkpoint inhibitors on kidney function over time: a single centre retrospective analysis

**DOI:** 10.1038/s44276-024-00081-7

**Published:** 2024-08-13

**Authors:** Benjamin M. P. Elyan, Michael K. Sullivan, James Hedley, Nicole De La Mata, Angela C. Webster, Balaji Venugopal, Rob J. Jones, Ninian N. Lang, Patrick B. Mark, Jennifer S. Lees

**Affiliations:** 1 School of Cardiovascular and Metabolic Health, College of Medical and Veterinary Life Sciences, University of Glasgow, Glasgow, UK; 2 NHS Greater Glasgow and Clyde, Glasgow, UK; 3 Collaborative Centre for Organ Donation Evidence, Sydney School of Public Health, Faculty of Medicine and Health, University of Sydney, Camperdown, New South Wales Australia; 4 Centre for Transplant and Renal Research, Westmead Hospital, Westmead, New South Wales Australia; 5 National Health and Medical Research Council Clinical Trials Centre, Faculty of Medicine and Health, University of Sydney, Camperdown, New South Wales Australia; 6 School of Cancer Sciences, College of Medical and Veterinary Life Sciences, University of Glasgow, Glasgow, UK

## Abstract

**Background:**

Drugs targeting angiogenesis and immunotherapy have transformed outcomes in renal cancer but may contribute to progressive kidney disease.

**Methods:**

We linked healthcare databases in the West of Scotland (spanning 2010–2020) to identify adults with renal cancer who received one or both classes of drugs. Over two years following initiation, estimated glomerular filtration rate (eGFR) slope was modelled using linear mixed-effects models. Additional renal outcomes used competing risk regression considering the competing risk of death.

**Results:**

Amongst 357 adults (62.5% male; median age 63.0 years, IQI 55.0–71.0), there was no significant change in eGFR (annual eGFR change +1.03 mL/min/1.73 m²/year, 95%CI −1.64 to +3.70), nor in subgroups of patients who had nephrectomy, metastatic cancer or an eGFR < 60 mL/min/1.73 m² prior to systemic therapy. A ≥ 40% decline in eGFR occurred in 82 people (23.0%) within one year of starting systemic therapy and was associated with pre-existing diabetes (subhazard ratio 1.89, 95%CI 1.05–3.41).

**Discussion:**

Anti-angiogenic and immune therapy had no substantial impact on the average change in eGFR but people with diabetes are at higher risk of clinically significant renal events. With appropriate monitoring, more widespread use of these agents in patients with renal impairment may be warranted.

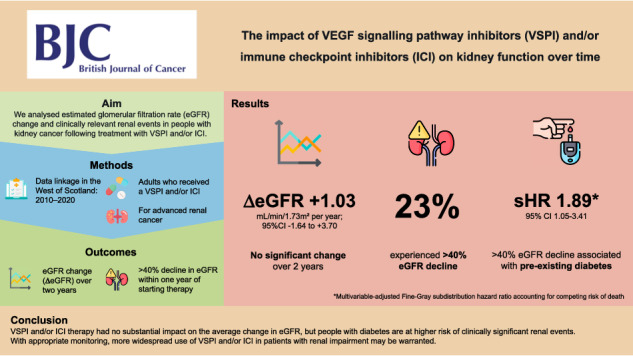

## Introduction

Long-term survival with well-controlled or cured cancer is rising, with cancer survival doubling in the last 50 years [1], in part due to the successful introduction of new systemic therapies into routine practice. VEGF-signalling pathway inhibitors (VSPI) and/or immune checkpoint inhibitors (ICI) now make up the first-line anti-cancer treatment for a range of cancers [2] and have transformed the outcomes of people with advanced renal cancer [3, 4]. They also carry a significant side effect profile including nephrotoxicity, hypertension and proteinuria [5, 6] which are independent risk factors for chronic kidney disease (CKD) [7, 8], cardiovascular events [9, 10] and mortality [11] in the general population. As cancer survivorship improves, consideration of longer-term consequences of cancer treatment, including renal and cardiometabolic health, becomes increasingly important.

VSPI and ICI therapy have become the mainstay of systemic treatment of advanced renal cancer, either as monotherapy or in combination with other agents [3, 12]. The reported incidence of adverse renal events varies but these are common and, in general, have a good prognosis for renal recovery [13]. Diagnosing renal adverse events from VSPI/ICI is challenging due to the lack of definitive diagnostic criteria and variability in the time of onset, which can be over a year after therapy initiation with some patients remaining on treatment for several years [13–15]. Kidney toxicity may be mediated by VSPI/ICI-induced effects including endothelial dysfunction, podocytopathies, glomerulonephritis, acute interstitial nephritis, thrombotic microangiopathy, hypertension, atherosclerosis and vasculitis [16–18]. Whilst hypertension is commonly observed as a side effect of VSPI therapy [19], documented renal safety events and their association with overall mortality is mainly limited to case reports, including those of irreversible kidney failure and nephrotic syndrome [20]. Systemic absorption of VSPI from intravitreal administration is associated with cases of accelerated hypertension, worsening proteinuria, glomerular disease, thrombotic microangiopathy, and possibly CKD [21].

Data regarding the longer-term risk of renal function decline from VSPI/ICI therapies are inconclusive [5, 15, 22]. Analysis for hard endpoints such as progression to ESKD over shorter follow-up duration, may miss a progressive decline in kidney function that could have a significant impact on quality of life or symptoms in people surviving longer after cancer diagnosis. Estimated glomerular filtration rate (eGFR) slope has been demonstrated as an important marker of kidney function decline and a surrogate marker for future kidney failure [23–25]. eGFR slope over 2–3 years is now routinely used as a critical outcome to delineate differences in the rate of kidney function decline for on- and off-treatment effects in clinical trials [26].

Outside the setting of VSPI/ICI therapy, the relationship between the development of cancer and kidney dysfunction is multifactorial. Prevalent cancer or CKD is associated with a higher risk of developing renal dysfunction than those without, including acute kidney injury (AKI) [27] and proteinuria. People with cancer have a high prevalence of CKD; furthermore, people with CKD have a higher incidence risk of certain cancers [28] and poorer survival [29] than those without CKD. Outcomes from cancer in the context of CKD may be affected by the influence of renal dysfunction upon anti-cancer treatment selection, duration, efficacy and safety [30].

Partial or total nephrectomy has become a pivotal management strategy in people with renal cancer [31, 32]. Nephrectomy, logically, causes a reduction in eGFR, but the elevated risk that this nephron loss poses to future risk of progressive CKD in people with renal cancer is unclear [33]. It appears that there are particular risk factors for developing adverse renal events following nephrectomy in the context of renal cancer, such as proteinuria [34, 35].

This study analyses the eGFR slope as well as clinically significant renal events, after the introduction of VSPI/ICI therapy in advanced renal cancer, and factors associated with a change in eGFR, renal events and all-cause mortality. By doing this, we aim to define the extent of renal risks associated with these therapies. We hypothesised that people treated with VSPI and/or ICI for renal cancer have a significant decline in eGFR over time and that there are higher risk groups of patients who are susceptible to a steeper eGFR decline.

## Methods

### Study design and data sources

We conducted a retrospective study using integrated data from comprehensive prescribing system, the electronic prescription system for anti-cancer therapies in the West of Scotland, and National Services Scotland SafeHaven databases. These datasets provide comprehensive patient information relating to all patients treated in NHS Greater Glasgow and Clyde (serving a population of 1.4 million), including laboratory data (SCI store), hospital records (SMR01), mortality records (National Records of Scotland – Deaths Data), cancer registry data (SMR06) and renal transplant or dialysis records (Strathclyde Electronic Renal Patient Record, SERPR, Vitalpulse, UK).

We included adults diagnosed with renal cancer who received VSPI or ICI, either as monotherapy or in combination as anti-cancer therapies within the Greater Glasgow and Clyde Health Board between January 2010 and December 2020. Adults with renal cancer were identified using the International Classification of Disease-10 (ICD-10: Version 2019) code C64 from linked cancer registry data from SMR06. People already receiving kidney replacement therapy at cancer incidence were excluded from the analysis. All people received their cancer treatment via the West of Scotland Cancer Network.

### Nephrectomy assessment

**Fig. 1:**
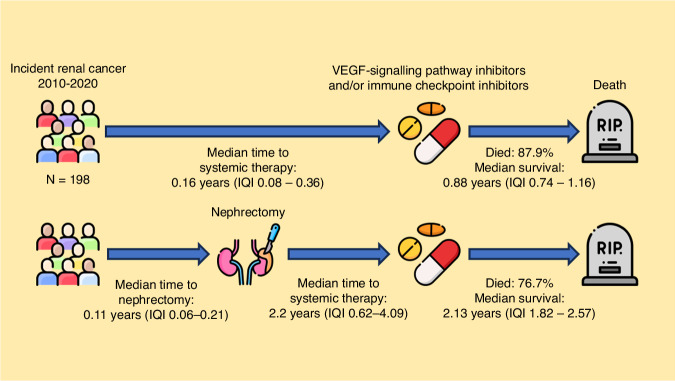
Cohort characteristics. Survival and follow-up duration between renal cancer diagnosis, nephrectomy and/or systemic therapy initiation.

People with nephrectomy or partial nephrectomy prior to systemic therapy were identified using ICD-10 codes and dates from SMR06 and SMR01 data. People with or without nephrectomy prior to systemic therapy were included (Fig. 1).

### Kidney function assessment

People who had at least one serum creatinine value available at any time before and two recordings after the initiation date of systemic therapy were included. eGFR was calculated using the CKD-EPI equation (2009) without race coefficient [36].

### Proteinuria assessment

Available urinary albumin-creatinine ratios (uACR) and protein–creatinine ratios (uPCR) from linked laboratory data were included from any time before and after the initiation of systemic therapy. If uPCR was the only available assessment of proteinuria, this was converted to uACR using a previously published conversion equation [37]. Pre-existing albuminuria before systemic therapy onset was categorised as uACR of > 3 mg/mmol (microscopic) or > 30 mg/mmol (macroscopic) [38].

### Metastatic cancer and comorbidity assessment

Metastatic cancer at the point of diagnosis was identified from ‘TNM’ staging data from the cancer registry SMR06 and defined as any patient having with metastatic (‘M’) cancer. People with prevalent diabetes prior to the initiation of systemic therapy were identified using ICD-10 codes E10 and E11 from linked hospitalisation data from SMR01.

### Renal outcomes

- eGFR slope: defined as change in eGFR within two years following the initiation of VSPI/ICI therapy.
- Death from renal cause: defined by ICD-10 codes N00-N19 as the primary cause of death in the death registry and SMR01.
- Progression to CKD Stage 5: defined as eGFR < 15 mL/min/1.73m^2^ at any time during follow-up sustained > 28 days.
- ESKD defined as renal transplantation or dialysis from SERPR.
- A ≥ 40% decline in eGFR from the baseline average eGFR within the first year of therapy [39].
- De-novo proteinuria: defined as normal uACR/uPCR results before treatment followed by elevated levels of proteinuria within one year of therapy [38].

### Overall survival

Overall survival was defined by time to death from any cause using death registry and SMR01 data. We reported median, 1-, 2- and 5-year survival. This was additionally analysed in subgroups of patients who did or did not have nephrectomy prior to the onset of systemic therapy.

### Statistical analysis

Descriptive statistics at baseline included counts and percentages for binary variables, while continuous variables were expressed as median (interquartile interval, IQI) or mean (standard deviation, SD).

eGFR slope was calculated for people who had two or more serum creatinine measures, within two years following the initiation of VSPI/ICI therapy. A linear mixed-effects model was used to analyse the slope of eGFR from the point of systemic therapy (commonly referred to as the total slope). This model included eGFR as the dependent variable and the time from the start of systemic therapy and the eGFR result (time difference) to allow for calculation of the change in eGFR per year from the point of systemic therapy. We employed random effects for the time difference variable for each patient to model distinct trajectories for each participant over time. We adjusted for age over 60 years at initiation of systemic therapy, sex, nephrectomy before systemic therapy, median eGFR < 60 mL/min/1.73m^2^ before systemic therapy and diabetes and metastatic cancer at diagnosis. These variables were selected for inclusion in the models due to biological plausibility as factors that might impact eGFR progression. They were included as independent binary variables and models assumed fixed effects. We accounted for individual specific variations by including a random effect for each patient. We included interaction terms: eGFR slope*nephrectomy prior to systemic therapy, eGFR slope*metastatic cancer and eGFR slope*median eGFR < 60 mL/min/1.73m^2^ before systemic therapy. The inclusion of these interaction terms in the model were tested using likelihood ratio test, using *p* < 0.05 as a significant improvement in model fit.

Cox proportional hazards models were used to analyse the clinically relevant associated factors of developing a ≥ 40% eGFR decline within the first year of systemic therapy and overall survival. We included univariable and multivariable models, which included the same relevant covariates as eGFR slope analysis. Fine and Gray subdistribution hazards were used to analyse the factors for developing a ≥ 40% eGFR decline within the first year of systemic therapy with a competing risk of death. Sensitivity analyses were conducted by therapy class using consistent analytical methods.

R software (version 4.3.2), employing packages tidyverse, finalfit, survminer, plot and lme4, was used for all analyses. The Model outputs and analysis will be available at publication (https://github.com/benelyan1/eGFR-slope-analysis).

## Results

### Baseline characteristics

**Fig. 2:**
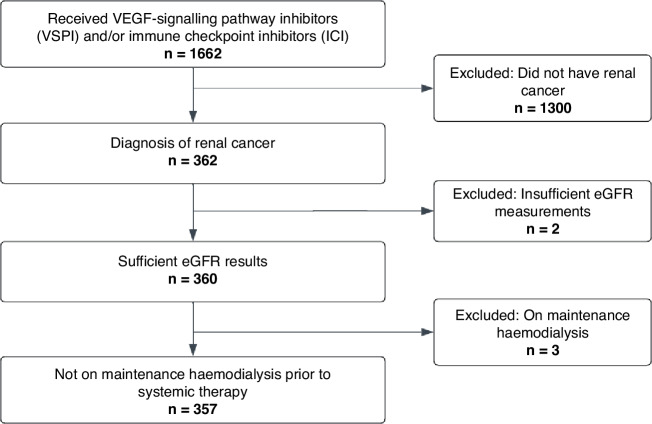
Flow chart. Patient inclusion flow chart.

We initially identified 1662 people who received VSPI and/or ICI, 362 of whom had renal cell cancer. We excluded two patients due to insufficient eGFR measurements and three who were on long-term dialysis at the start of systemic therapy. We included 357 patients (62.5% male; median age 63.0 years, IQI 55.0–71.0) with renal cell cancer who had been treated with VSPI and/or ICI (Table 1). (Fig. 2).

**Table 1:** Baseline characteristics of the cohort and split by nephrectomy pre and post systemic therapy.

Characteristic		Nephrectomy before systemic therapy	No nephrectomy before systemic therapy	Overall	*p*
Total: *n* (%)		153 (42.9)	204 (57.1)	357 (100%)	
Age at systemic therapy: median (IQI)		62.0 (55.0–71.0)	64.0 (55.0–70.2)	63.0 (55.0–71.0)	0.536
Sex: *n* (%)	Male	100 (65.4)	127 (62.3)	227 (63.6)	0.623
	Female	53 (34.6)	77 (37.7)	130 (36.4)	
Median eGFR before systemic therapy: median (IQI)		70.6 (56.6–84.9)	79.0 (61.4–96.0)	74.6 (58.3–91.9)	<0.001
Microalbuminuria before systemic therapy: *n* (%)	Yes	35 (57.4)	35 (55.6)	70 (56.5)	0.981
	No	26 (42.6)	28 (44.4)	54 (43.5)	
Macroalbuminuria before systemic therapy: *n* (%)	Yes	6 (9.8)	5 (7.9)	11 (8.9)	0.955
	No	55 (90.2)	58 (92.1)	113 (91.1)	
Regime class: *n* (%)	ICI monotherapy	*	7 (3.4)	*	0.081
	VSPI monotherapy	138 (90.2)	164 (80.4)	302 (84.6)	
	Dual ICI	*	14 (6.9)	*	
	VSPI + ICI combination therapy	8 (5.2)	19 (9.3)	27 (7.6)	
Metastatic cancer at diagnosis: *n* (%)	Yes	25 (16.3)	94 (46.1)	119 (33.3)	<0.001
	No	128 (83.7)	110 (53.9)	238 (66.7)	
Diabetes before systemic therapy: *n* (%)	Yes	21 (13.7)	29 (14.2)	50 (14.0)	0.999
	No	132 (86.3)	165 (85.8)	307 (86.0)	

Proportions of micro- and macroalbuminuria are reported for those that had quantification by urinary albumin or protein–creatinine ratio. *n* number, *IQI* Interquartile interval.

*In this table, data points and totals representing fewer than 5 records have been suppressed and replaced with an asterisk to protect patient confidentiality.

Median follow-up from start of systemic therapy to the last eGFR measurement or death was 1.35 years (IQI 0.50–2.61) and each person had a median of 25 eGFR measurements (IQI 13–38) after systemic therapy. VSPI monotherapy was the predominant choice of systemic therapy (86.0%) and was given for a median of five cycles of treatment (IQI 2–9). For the 150 (42.9%) people who had nephrectomy, the median time from nephrectomy to starting systemic therapy was 2.2 years (IQI 0.62–4.09) (Fig. 1).

Prior to systemic therapy, 92 people (25.8%) had an eGFR <60 mL/min/1.73m^2^ and 4 people (1.1%) had an eGFR < 30 mL/min/1.73m^2^. People who had nephrectomy (42.9%) prior to systemic therapy had a lower pre-treatment median eGFR (70.4 vs 78.9 mL/min/1.73m^2^, *p* < 0.001) and fewer had metastatic cancer at diagnosis (16.3% vs 46.1%, *p* < 0.001). There was considerable overlap between the people who had one or more of nephrectomy prior to systemic therapy, metastatic cancer at the point of diagnosis and an eGFR < 60 mL/min/1.73m^2^ (Supplementary fig. S1). Diabetes was present in 50 people (14.0%). uACR or uPCR values were available for 124 people (34.7%). Of these, 70 (56.5%) had evidence of microalbuminuria before commencing therapy and 11 (8.9%) had macroalbuminuria.

### eGFR slope analysis

**Fig. 3:**
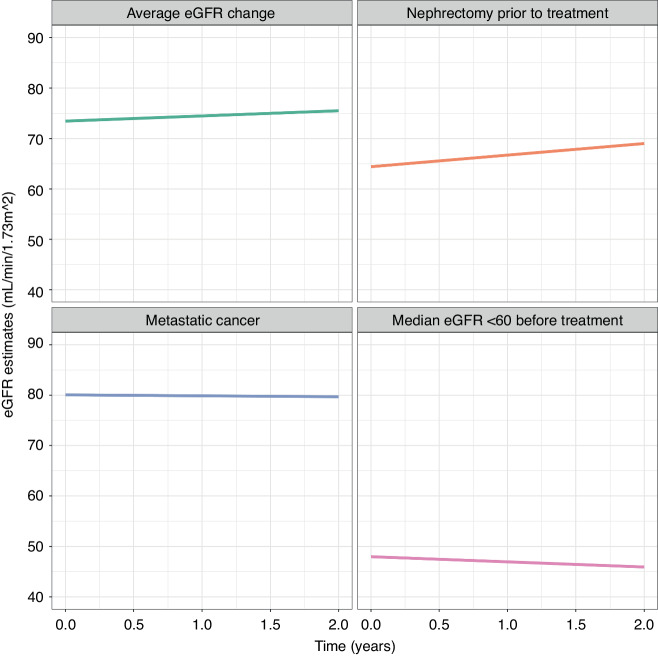
eGFR slope estimates over time of people with renal cancer treated with VEGF-signalling pathway inhibitors and/or immune checkpoint inhibitors using the linear mixed-effects model from the point of systemic therapy to 2 years from follow-up. Demonstrated as the average eGFR change of the of the group, and specifically for people who had nephrectomy prior to systemic therapy, metastatic cancer at the point of diagnosis and an average eGFR <60 ml/min/1.73m^2^ before systemic therapy.

On average, there was no significant change in eGFR per year in people with renal cancer who had VSPI/ICI treatment ( + 1.03 mL/min/1.73m^2^/year; Table 2), nor in subgroups of people who had nephrectomy prior to systemic therapy ( + 2.30 mL/min/1.73m^2^/year), with metastatic cancer at the point of diagnosis (−0.18 mL/min/1.73m^2^/year), or with an eGFR < 60 mL/min/1.73m^2^ before systemic therapy (−1.02 mL/min/1.73m^2^/year; Fig. 3). A sensitivity analysis of people who received only VSPI monotherapy revealed similar results. There were insufficient numbers of people who received ICI monotherapy to perform a sensitivity analysis for this group.

**Table 2:** Coefficient of change in eGFR per year in the two years following initiation of systemic therapy (average change in eGFR per year and for specific patient groups per year).

Patient group	Coefficient of eGFR change per year (mL/min/1.73m^2^)	95% CI: lower	95% CI: upper	*p*-value
Whole cohort (unadjusted)	+1.03	−1.64	+3.70	0.23
Nephrectomy prior to systemic therapy	+2.30	−1.66	+6.26	0.52
Metastatic cancer at the point of diagnosis	−0.18	−5.25	+4.89	0.648
Average eGFR < 60 prior to systemic therapy	−1.02	−6.55	+4.50	0.473

### Renal events analysis

**Fig. 4:**
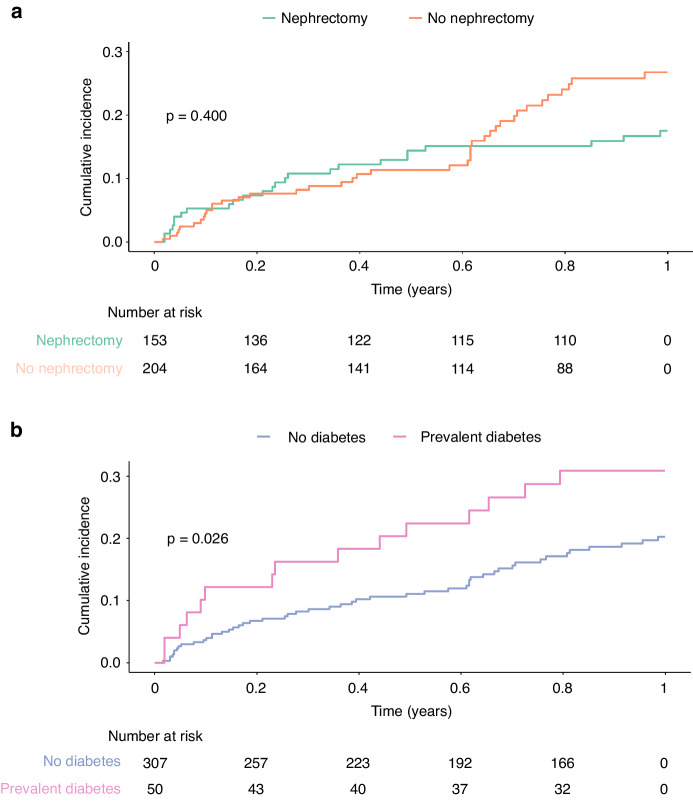
Cumulative incidence curves for a ≥ 40% decline of eGFR from the baseline eGFR at the time of initiation of systemic therapy. The curves are stratified by people who (**a**) did or did not have nephrectomy prior to systemic therapy and (**b**) by those who did or did not have prevalent diabetes.

During the follow-up period, 82 (23.0%) people experienced a ≥ 40% decline in eGFR from baseline (Fig. 4). The cumulative incidence of this ≥40% decline in eGFR did not differ significantly between those that did or did not have nephrectomy prior to systemic therapy (*p* = 0.13).

In univariable analysis, diabetes was associated with a 1.72-fold increased risk of ≥40% eGFR decline one year post systemic therapy (95% CI 1.01–2.94, *p* = 0.046), but this association was attenuated after multivariable adjustment (HR 1.70, 95% CI 0.99–2.92, *p* = 0.052). Competing risk analysis (Table 3) identified that diabetes diagnosis was the only covariate that demonstrated statistically significant associations of eGFR decline of ≥40% (subhazard ratio 1.89, 95% CI 1.05–3.41, *p* = 0.035). A sensitivity analysis of the people who received VSPI monotherapy revealed similar results and there were insufficient numbers of people who received ICI monotherapy to perform a sensitivity analysis for this group.

**Table 3:** Hazard of eGFR decline of 40% from baseline eGFR within 1 year from the onset of systemic therapy.

Dependent: 40% eGFR decline within 1 year of systemic therapy		Total (*n*)	HR (CPH univariable)	HR (CPH multivariable)	HR (Competing risks multivariable)
Age at treatment	Median (IQR)	63.0 (55.0–71.0)	1.01 (0.99v1.03, *p* = 0.379)	1.01 (0.99–1.04, *p* = 0.228)	1.01 (0.99–1.03, *p* = 0.380)
Nephrectomy prior to systemic therapy	Yes	153	-	-	-
	No	204	0.87 (0.56–1.34, *p* = 0.517)	0.90 (0.57–1.43, *p* = 0.663)	1.22 (0.71–2.12, *p* = 0.470)
Sex	Male	227	-	-	-
	Female	130	0.97 (0.62–1.53, *p* = 0.896)	0.99 (0.63–1.56, *p* = 0.966)	0.91 (0.54–1.53, *p* = 0.720)
Average eGFR < 60 prior to systemic therapy	Yes	96	-	-	-
	No	261	1.08 (0.66–1.77, *p* = 0.762)	1.28 (0.75–2.16, *p* = 0.363)	0.94 (0.54–1.63, *p* = 0.840)
Diabetes	No	307	-	-	-
	Yes	50	1.77 (1.04–3.01, *p* = 0.037)	1.76 (1.03–3.01, *p* = 0.040)	2.01 (1.11–3.62, *p* = 0.021)
Metastatic cancer	No	238	-	-	-
	Yes	119	0.70 (0.43–1.15, *p* = 0.158)	0.72 (0.43–1.22, *p* = 0.225)	0.87 (0.49–1.55, *p* = 0.640)

This is demonstrated as univariable and multivariable cox proportional hazards (CPH) for developing this decline. Fine and Grey subdistribution hazards for developing this decline with a competing risk of death (competing risks multivariable). Multivariable adjustment for all the included covariates.

Four people (1.1%) progressed to a sustained eGFR < 15 mL/min/1.73m^2^ during follow-up. One of these was established on long-term kidney replacement therapy. None of the people treated with VSPI/ICI who died during follow up had renal failure listed as the primary cause of death.

### De-novo proteinuria within 1 year of systemic therapy

Proteinuria quantification was poorly documented. Of the 287 people who did not have proteinuria prior to systemic therapy, proteinuria was quantified within one year of therapy in 75 (27.1%) people. De-novo microscopic albuminuria developed in 23 people (8.3% of those who had quantification), and de-novo macroscopic albuminuria developed 13 people (4.7%).

Patients who had not undergone nephrectomy had higher risk of developing microscopic albuminuria within one year of systemic therapy than those who had not had a nephrectomy (subhazard ratio 4.72, 95% CI 1.14–19.44, *p* = 0.032). The other covariates did not reach statistical significance. No factors were independently associated with the development of macroalbuminuria.

### Overall survival

During follow-up, 297 (83.2%) people died. Median survival was 1.36 years (IQI 1.11–1.75 years). Overall survival at one year was 58.3% (95% CI: 53.4–63.6%), two years 39.6% (95% CI: 34.8–45.0%), and five years 17.2% (95% CI: 13.3–22.2%). For people who had nephrectomy prior to systemic therapy, the median survival was longer: 2.13 years (IQI 1.82–2.57) than people who did not have nephrectomy: 0.88 years (IQI 0.74–1.16). The difference in median survival between the groups was statistically significant (log-rank test: Chi-Square = 19.6, *p* < 0.001, Supplementary fig. S2).

Metastatic cancer at the point of diagnosis and absence of nephrectomy prior to systemic therapy were associated with increased hazards of death on univariable analysis (*p* < 0.001). These associations remained significant after adjusting for age, sex, metastatic cancer, nephrectomy and median baseline eGFR < 60 mL/min/1.73m^2^ (Table 4, Supplementary fig. S3: Adjusted survival HR for no nephrectomy: 1.51 [95% CI: 1.18–1.94, *p* = 0.001]; Adjusted survival HR for metastatic cancer: 1.77 [95% CI: 1.36–2.29, *p* < 0.001]). A ≥ 40% acute or chronic decline in eGFR was not associated with increased hazards of death on univariable or multivariable analysis (Adjusted survival HR: 1.11 [0.85–1.46, *p* = 0.443]).

**Table 4:** Hazards of death from the onset of systemic therapy.

Dependent: Hazards of death		all	Total	HR (CPH univariable)	HR (CPH multivariable)
Age > 60 at initiation of systemic therapy	No	152 (42.6)	152 (42.6)	-	-
	Yes	205 (57.4)	205 (57.4)	0.95 (0.75–1.19, *p* = 0.660)	0.89 (0.71–1.12, *p* = 0.317)
Nephrectomy prior to systemic therapy	Yes	153 (42.9)	153 (42.9)	-	-
	No	204 (57.1)	204 (57.1)	1.73 (1.37–2.19, *p* < 0.001)	1.53 (1.20–1.96, *p* = 0.001)
Sex	Male	227 (63.6)	227 (63.6)	-	-
	Female	130 (36.4)	130 (36.4)	1.13 (0.89–1.43, *p* = 0.311)	1.14 (0.90–1.44, *p* = 0.273)
40% eGFR decline within 1 year of systemic therapy	No	275 (77.0)	275 (77.0)	-	-
	Yes	82 (23.0)	82 (23.0)	1.06 (0.81–1.39, *p* = 0.676)	1.11 (0.85–1.46, *p* = 0.443)
Metastatic cancer	No	238 (66.7)	238 (66.7)	-	-
	Yes	119 (33.3)	119 (33.3)	1.84 (1.45–2.33, *p* < 0.001)	1.62 (1.26–2.08, *p* < 0.001)

This is demonstrated as univariable, multivariable cox proportional hazards (CPH) for death. Multivariable adjustment for all the included covariates.

## Discussion

This study explores the determinants of kidney function in people with renal cancer treated with VSPI and/or ICI. Our findings are generally reassuring for the average change in kidney function after the initiation of VSPI and/or ICI but shed light on several critical aspects related to renal outcomes associated with these important anti-cancer drugs.

The total cohort demonstrated no significant impact in yearly change of eGFR. Notably, this was true for specific subgroups including people with a history of nephrectomy, with metastatic disease at diagnosis or an average eGFR < 60 mL/min/1.73m^2^ prior to systemic therapy. The impact of these therapies on kidney function in the medium to long term had been unclear despite the justifiable concerns regarding the development of known risk factors for progressive CKD (e.g., hypertension) following their initiation. Whilst our findings are reassuring with regards to kidney function decline at least within the first two years of commencing treatment, they also suggest a complex interplay between cancer progression, survivorship, systemic therapy, management strategies and kidney function measurement in this cohort.

The positive trends in eGFR are likely to represent a fall in serum creatinine due to factors other than kidney function improvement, such as sarcopenia, weight loss and dietary changes. Accurate measurements of kidney function in people with cancer is crucial but often challenging. Inaccurate measurements of eGFR have been shown previously to associate with higher rates of drug-induced toxicity [40] and eligibility for systemic treatments [41]. Cockcroft Gault measurements of creatinine clearance (cg-CrCl) are commonly used in practice for drug dosing, despite a lack of validation in a cancer cohort [42]. Alternative markers of glomerular filtration (such as cystatin C, panel eGFR or measured GFR) [43, 44] and appropriate consideration of the role of tubular creatinine secretion [45] after VSPI/ICI treatment might have yielded different results.

We observed a ≥ 40% decline in eGFR within the first year of systemic therapy in almost a quarter of the participants, and a very small proportion (1.1%) developed a persistent drop in eGFR to < 15 mL/min/1.73m^2^ or ESKD. The reported rate of nephritis or ‘raised creatinine’ is lower in randomised controlled trials of VSPI [46] and/or ICI [47] as anti-cancer treatments. Whilst it appears that the overall trend of kidney function does not decline, people commonly experience clinically important renal events following treatment initiation and there may be people who are at higher risk of experiencing these events. Diabetes emerged as a key risk factor and, although this association was attenuated after multivariable adjustment, a mechanistically plausible trend to a link between diabetes and worse renal outcomes remained. Whilst a ≥ 40% decline in eGFR within the first year of systemic therapy was not associated with an increased hazard of death, a competing risk multivariable regression analysis censoring for death is an important analysis to consider in this group with a high mortality rate. Diabetes was associated with an increased risk of ≥ 40% decline in eGFR within the first year of systemic therapy, however, diabetes has previously been associated with an overall increased risk of acute kidney injury compared with those without diabetes [48]. The number of people developing eGFR < 15 ml/min/1.73^2^ or ESKD was too small to make further analyses, but further supports the need to study renal-specific implications of these therapies, particularly as their indications broaden to include the treatment of a broader range solid organ tumours and at increasingly early stages [2].

The representation of people with eGFR < 30 mL/min/1.73m^2^ prior to the initiation of treatment in this cohort was low. This is despite the high prevalence of people with cancer who have co-existing CKD [49], and the large proportion of people in this cohort who had partial or total nephrectomy as part of their cancer management. The under-representation of patients with more advanced CKD suggests that people with CKD may not be treated with these anti-cancer therapies on the basis of reduced kidney function. Our data suggest that exclusion from therapy on this basis may not be justified. People with CKD are under-represented in clinical trials [50] which may also contribute to relative underuse of these drugs in patients with CKD. However, we did note that three people were on maintenance haemodialysis when treated with VSPI/ICI. People with an eGFR < 60 mL/min/1.73m^2^ prior to systemic therapy did not demonstrate an excessive decline in eGFR slope per year or high rates of a decline of eGFR by ≥40% from baseline within a year. Whilst this population may be prone to selection bias, it does suggest that renal side effects are not an overwhelming issue in this population.

Additionally, 1 in 13 people who had no evidence of proteinuria prior to systemic therapy developed microscopic albuminuria within the first year of systemic therapy although the vast majority did not have proteinuria tested following systemic therapy initiation. The absence of nephrectomy prior to systemic therapy emerged as a significant risk factor for developing de-novo microscopic albuminuria, potentially overlapping with other factors that may be implicated in the decision not to have operative management of the cancer. Initial nephrectomy in patients who present with metastatic disease remains controversial and is generally reserved for those with more favourable prognostic features who may be less likely to suffer renal consequences of advanced cancer. Furthermore, patients who undergo nephrectomy with curative intent but subsequently relapse with metastatic disease have better prognosis and, similarly, may be less likely to suffer renal events on treatment. In general, baseline and subsequent proteinuria was poorly documented in this cohort despite it being well established as a known side effect [51]. However, the long-term implications of developing proteinuria in this setting remain poorly understood.

The clinical significance of the nephron loss following nephrectomy in the context of advanced renal cancer is unclear and complex, due to the impact of cancer on kidney function and some shared risk factors for renal cancer and progressive CKD such as smoking and genetic conditions [30]. There appear to be understandable but important differences in the risk of reduced kidney function between radical nephrectomy and partial nephrectomy [33]. In the context of kidney donation for transplantation, nephrectomy does appear to result in an elevated risk of proteinuria [52] and ESKD [53], despite a significant compensatory rise in eGFR following surgery [54]. Importantly, our data demonstrate that people who had nephrectomy were not a greater risk for average eGFR decline.

While our study provides valuable insights into the renal-specific outcomes of VSPI/ICI therapy in people with renal cancer, it is essential to acknowledge its limitations. First, the high death rate of this cohort may impair the application of these findings to other contemporary cohorts of patients who may have better longer-term survival and longer exposure to treatment. Second, we could not comment on measured kidney function or alternative measures of kidney function as—in keeping with widespread clinical practice—we were limited to creatinine-based measures of eGFR. We have reported eGFR indexed to body surface area (BSA) of 1.73m^2^, though eGFR non-indexed to BSA may be preferable for drug dosing in situations where BSA is substantially different from the reference value (as may be seen in cancer-associated cachexia). We did not have longitudinal height and weight data to report non-indexed values. Third, the small numbers who had quantification of proteinuria limit the ability to draw conclusions about the entire cohort and analysis of association with outcomes. We did not have data on dipstick urinalysis assessment which further impairs our ability to draw conclusions about the risk of proteinuria. Fourth, we did not have a comparison cohort of patients with renal cancer who did not receive VSPI or ICI, so were unable to dissect the relative contributions of treatment and the cancer itself on the observed renal events. Fifth, we had insufficient numbers to compare combination treatments to monotherapy in our sensitivity analysis. Finally, our cohort was from a single centre and participants were predominantly Caucasian, which may limit generalisability to other populations.

## Conclusion

VSPI and ICIs offer people with renal cancer significant improvement in survivorship. Despite case series and prescribing guidelines highlighting adverse impact of VSPI/ICI therapy on renal function, our real-world data on the effect of VSPI/ICI therapy on renal function demonstrate that there is no significant impact on the average change in eGFR but highlights that some groups are at higher risk of clinically significant renal events, such as people with diabetes. Our data suggest that people with prior renal dysfunction may, potentially inappropriately, be denied access to life-prolonging anti-cancer therapy. Further investigation into appropriate renal risk stratification and optimal surveillance strategies is required for people with cancer treated with VSPI and/or ICI.

## Supplementary information

Supplementary appendix

## Data Availability

Data used for this study are available through the West of Scotland ChemoCare and West of Scotland SafeHaven network. The Model outputs and analysis will be made available at the time of publication (https://github.com/benelyan1/eGFR-slope-analysis).
