# Supplementary material for: The impact of VEGF signalling pathway inhibitors and/or immune checkpoint inhibitors on kidney function over time: a single centre retrospective analysis
Source: BJC Rep. 2024 Aug 13;2:57. doi: 10.1038/s44276-024-00081-7 (PMC11523961; doi:10.1038/s44276-024-00081-7)
Supplement: Supplementary file 1 — Supplementary appendix [file 44276_2024_81_MOESM1_ESM.docx]

## Supplementary figure S1

Representation and overlap of nephrectomy before systemic therapy, metastatic cancer at the point of diagnosis and an eGFR <60 before systemic therapy.


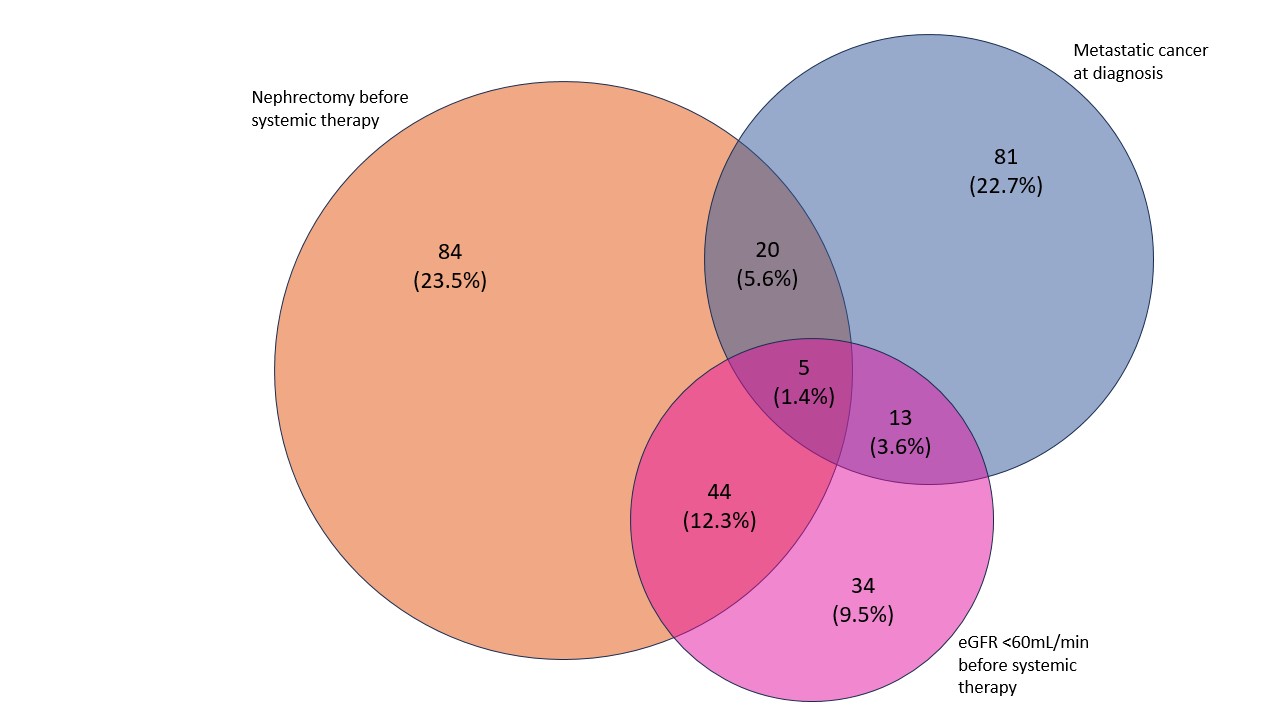


## Supplementary figure S2

Unadjusted survival curves of overall survival in the total population. The curves are stratified by nephrectomy status prior to systemic therapy. This plot has been curtailed at 5 years due to low patient survival beyond 5 years.





## Supplementary figure S3

Plot demonstrating the hazards of death in the total population, adjusted for nephrectomy prior to systemic therapy, age, sex and median eGFR <60mL/min/1.73m^2^ prior to systemic therapy.
